# Oral supplementation with selected *Lactobacillus acidophilus* triggers IL-17-dependent innate defense response, activation of innate lymphoid cells type 3 and improves colitis

**DOI:** 10.1038/s41598-022-21643-0

**Published:** 2022-10-20

**Authors:** Jiří Hrdý, Aurélie Couturier-Maillard, Denise Boutillier, Carmen Lapadatescu, Philippe Blanc, Jan Procházka, Bruno Pot, Bernhard Ryffel, Corinne Grangette, Mathias Chamaillard

**Affiliations:** 1 Univ. Lille, CNRS, Inserm, CHU Lille, Institut Pasteur de Lille, U1019-UMR 9017-CIIL-Centre d’Infection et d’Immunité de Lille, 59000 Lille, France; 2 INEM-UMR7355, Molecular Immunology, Institute of Infectious Diseases and Molecular Medicine (IDM), and Infectious Diseases, University and CNRS, Orléans, France; 3 Bioprox, 7 rue Aristide Briand, 92300 Levallois-Perret, France; 4 Institute of Molecular Genetics, Czech Centre for Phenogenomics, Czech Academy of Sciences, 252 50 Vestec, Czech Republic; 5 Division of Immunology and South African Medical Research Council (SAMRC) Immunology, Department of Clinical Immunology, Faculty of Health Sciences, University of Cape Town, Anzio Road, Observatory, 7925 Cape Town, RSA; 6 Present Address: Institute of Immunology and Microbiology, First Faculty of Medicine, Charles University, Prague, Czech Republic; 7 Present Address: Research Group of Industrial Microbiology and Food Biotechnology, Faculty of Sciences and Bioengineering Sciences, Vrije Universiteit Brussel, Brussels, Belgium; 8 Present Address: Univ. Lille, Inserm, U1003, 59000 Lille, France

**Keywords:** Cell biology, Immunology, Microbiology, Gastroenterology

## Abstract

Live biotherapeutic products constitute an emerging therapeutic approach to prevent or treat inflammatory bowel diseases. *Lactobacillus acidophilus* is a constituent of the human microbiota with probiotic potential, that is illustrated by improvement of intestinal inflammation and antimicrobial activity against several pathogens. In this study, we evaluated the immunomodulatory properties of the *L. acidophilus* strain BIO5768 at steady state and upon acute inflammation. Supplementation of naïve mice with BIO5768 heightened the transcript level of some IL-17 target genes encoding for protein with microbicidal activity independently of NOD2 signaling. Of these, the BIO5768-induced expression of Angiogenin-4 was blunted in monocolonized mice that are deficient for the receptor of IL-17 (but not for NOD2). Interestingly, priming of bone marrow derived dendritic cells by BIO5768 enhanced their ability to support the secretion of IL-17 by CD4^+^ T cells. Equally of importance, the production of IL-22 by type 3 innate lymphoid cells is concomitantly heightened in response to BIO5768. When administered alone or in combination with *Bifidobacterium animalis* spp. *lactis* BIO5764 and *Limosilactobacillus reuteri*, BIO5768 was able to alleviate at least partially intestinal inflammation induced by *Citrobacter rodentium* infection. Furthermore, BIO5768 was also able to improve colitis induced by 2,4,6-trinitrobenzene sulfonic acid (TNBS). In conclusion, we identify a new potential probiotic strain for the management of inflammatory bowel diseases, and provide some insights into its IL-17-dependent and independent mode of action.

## Introduction

Crohn’s disease (CD) is traditionally characterized by impaired dendritic cells function^1^ and the development of transmural inflammatory lesions leading to progressive destruction of the intestinal wall. In Europe, the combined prevalence is about 250–300 cases per 100,000 inhabitants. Several epidemiological and experimental evidences indicate that incidence rates in emerging countries are rising due to the influence of many gene-environmental interactions, such as, amongst others, NOD2 signaling and tobacco smoking^2,3^. While the lifetime therapeutic management of the disease is far from optimal, CD alters patient’s ability to work and their social interaction in various ways. Invasive surgery and bowel resection is required in about two-thirds of CD patients, although it does not cure the disease. Consequently, the annual economic burden of medical care of CD patients ranges between 2.1 and 16.7 billion € in Europe with about 2.2 million people affected^4^.

The gut microbiota plays a crucial role in many physiological processes from the earliest days of life, including the maturation of intestinal immunity. Recent progress in high-throughput sequencing revealed that lowered bacterial diversity is commonly observed in the gut microbiota of CD patients^5,6^. Specifically, a decreased prevalence of the anti-inflammatory *Faecalibacterium prausnitzii* was associated with a higher risk of postoperative recurrence of ileal CD^7^. Inappropriate interaction between *F. prausnitzii* and the mucosal immune system is thereby thought to progressively impairs the integrity of the intestinal barrier and subsequently influence disease initiation and progression^8,9^. During the early postnatal period when the gut associated lymphoid tissue is developing, it has now been demonstrated that both interleukin-17 (IL-17) and interleukin-22 (IL-22) may shape the gut microbiota composition and promote the integrity of the intestinal barrier^10^. This said, both IL-17 and IL-22 needs to be tightly regulated to prevent unwarranted tissue damage and epithelial cell proliferation. At steady state, the major source of IL-17 and IL-22 are CD4-expressing T cells and the type 3 innate lymphoid cell (ILC3) respectively, defect of which may result in dysbiosis and increased susceptibility to intestinal inflammation^11–13^. Under normal conditions, ILC3 is induced by bacterial metabolites such as SCFA or tryptophan metabolites. Pleiotropism in T_h_17-associated responses may at least be attributed to IL-22. The aforementioned protective effect of IL-17 and IL-22 mainly relies on the downstream secretion of anti-microbial peptides (AMPs) by intestinal epithelial cells. Even if a matter of ongoing debate concerns the precise epithelial cell type that is targeted by IL-17 and IL-22, we and other postulated that the treatment of IBD may be improved with therapeutic agents that reinforce IL-17 and IL-22-mediated intestinal barrier function.

Supplementation with health-promoting probiotics is becoming particularly attractive not only to reconstitute the diversity and the functionality of patient’s microbiome but also to counteract the exaggerated inflammatory responses. While probiotics showed successful impact against ulcerative colitis, none of the reported clinical trials with probiotics proved some efficacy in CD^14–17^. A possible explanation for such inefficiency is that their mode of action may rely on certain genes that are found mutated in CD patients. In agreement with this hypothesis, we provided evidence that the protective capacity of a selected strain of *Ligilactobacillus salivarius* Ls33 (*formerly known as Lactobacillus salivarius* Ls33) requires an intact nucleotide-binding oligomerization domain 2 (NOD2) signaling^18^, mutations of which occur in more than one third of CD patients in Europe and North America. We then performed a comprehensive screening of the Bioprox probiotic collection for identifying strains that are exhibiting anti-inflammatory and antimicrobial abilities independently of NOD2 signaling. This led us to identify a *Lactobacillus acidophilus* strain able to induce IL-17-dependent antimicrobial responses independently of NOD2. This rod-shaped strain BIO5768 that produces lactic acid through the fermentation of carbohydrates was originally isolated from the human gastrointestinal tract and is produced and commercialized as dietary supplements by the society Bioprox Healthcare.

In the current study, we report that supplementation with *L. acidophilus* BIO5768 enhanced the activity of CD4-expressing T cells and type 3 innate lymphoid cells (ILC3), that are known to play an essential role in the maintenance of the barrier function and tissue repair^16,19^. ILC3 is a heterogeneous population consisting of three subpopulations: NCR^+^ ILC3, NCR^-^ ILC3 and LTi. Specifically, we tested their capacity to modulate the expression of antimicrobial peptides (AMPs) and the secretion of IL-17 both in vitro and in vivo. Of these, the expression of Angiogenin 4 that is secreted by Paneth cells and Goblet cells into the gut lumen was enhanced in response to BIO5768 in an IL-17-dependent (but not NOD2-independent) manner. Equally of importance, its ability to induce the secretion of additional AMPs (such as Defa4, Defb2, Reg3b and Reg3g) was shown to be independent of IL-17. Given that BIO5768 exhibited a different mode of action than the previously studied strains *Bifidobacterium animalis* spp. *lactis* BIO5764 (referred herein as BlO5764) and *Limosilactobacillus reuteri* BIO5454 (formerly known as *Lactobacillus reuteri* BIO5454, referred herein as BIO5454)^20^, we compared its anti-inflammatory capacity in vivo when administered alone or in combination, by using a *Citrobacter rodentium* infection model. The anti-inflammatory property of BIO5768 was then confirmed by making use of a TNBS colitis model.

## Results

### *L. acidophilus* BIO5768 heightens IL-22 production by type 3 innate lymphoid cells and maturation of dendritic cell that supports IL-17 by CD4-expressing T cells

**Figure 1:**
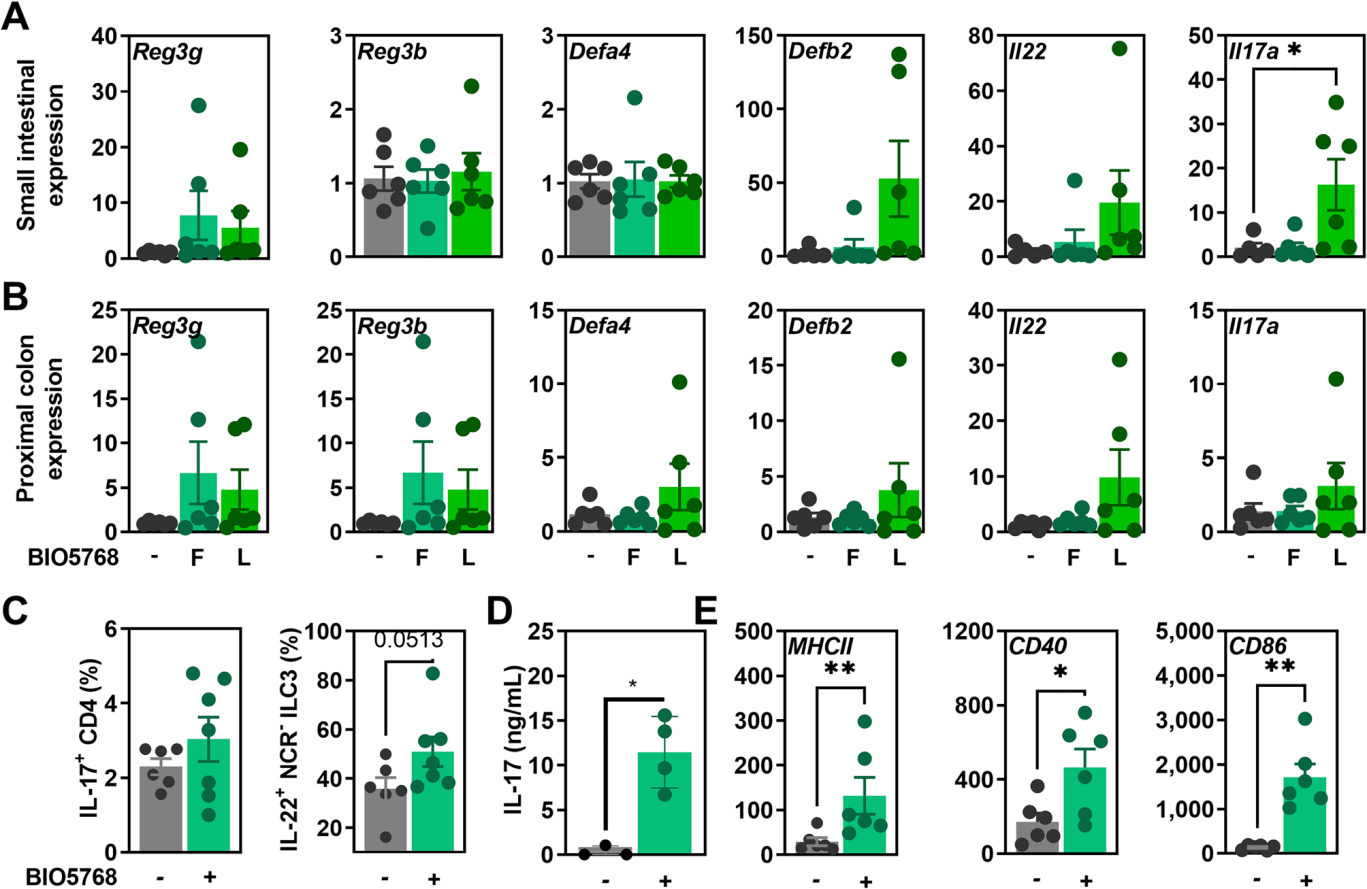
Capacity of the *L. acidophilus* BIO5768 to induce the expression of antimicrobial genes downstream of IL-17 and IL-22 and to activate bone marrow derived dendritic cells (BMDC) for supporting production of IL-17- and IL-22. Gene expression by qRT-PCR analysis of *Reg3g, Reg3b*, *Defa4*, *Defb2, Il22* and *Il17a* (**A**) in the small intestine or (**B**) in the proximal colon of BALB/c mice after 5 days of either fresh (F; n = 6) or lyophilized (L; n = 6) BIO5768 supplementation when compared to controls (n = 6). Results are expressed as relative gene expressions (2^−∆∆ct^) values, by comparing the PCR cycle thresholds (Ct) for the gene of interest and for the house keeping gene β-actin (*bact*), compared with control mice. (**C**) Percentage of IL-17-producing CD4 T cells and of IL-22 producing NCR- ILC3 within mesenteric lymph nodes of mice that were supplemented with BIO5768 (n = 7) when compared to control animals (n = 6), as determined by flow cytometry. (**D**) Measurement of IL17 production by ELISA in the supernatant collected after 24 h of coculture between untreated or BIO5768-stimulated BMDCs and naïve CD4 T cells that were sorted by magnetic beads. (**E**) Comparison of MFI (Mean of Fluorescence Intensity) of activation markers MHCII, CD40 and CD86 in untreated or BIO5768-stimulated BMDCs. Representative results of 6 independent in vitro experiments are shown (bars represent mean ± SEM). * p < 0.05; **p < 0.01; ***p < 0.001.

We first evaluated the capacity of either freshly cultured (F) or lyophilized (L) BIO5768 to regulate the expression of Th17 cytokines and their antimicrobial target genes in the small and large intestine of mice. Oral supplementation with lyophilized (but not freshly cultured) BIO5768 significantly increased the expression of *Il17a* (p < 0.05) and to a lower extent of *Il22* (p = 0.0519) in the small intestine Fig. 1A). In agreement, the expression of the *Defb2* gene significantly correlated with its upstream regulator IL-22 (Spearman r 0.7637, P value two tailed = 0.0034). Similar results were observed in the colon, but the difference did not reach significance (Fig. 1B). These result suggested that BIO5768 may preferentially adhere to the small intestine for strengthening the activity of Th17 and ILC3 cells at steady state. This led us to evaluate the production of IL-17 and IL-22 by their major cellular sources within the draining mesenteric lymph nodes (MLN). In agreement with our qRT-PCR analysis, flow cytometry analysis revealed an increased number of IL-22-producing Natural Cytotoxicity Receptor negative (NCR-) ILC3 in the MLN of mice supplemented by BIO5768 (Fig. 1C and Figs. S1 and S2). By contrast, a similar frequency of either IL-17-producing CD4 T cells or IL-22-producing NCR + ILC3 cells was observed in the MLN of treated and untreated mice (Fig. S3). Furthermore, BIO5768 was not able to expand the number of regulatory T cells (Fig. S3). We then evaluated the capacity of the BIO5768 strain to induce the maturation of bone marrow derived dendritic cells (BMDCs) that play a key role in the induction of cellular immunity and polarization of protective T helper cell subsets. To this end, BMDCs from BALB/c mice were stimulated by BIO5768 for 24 h before being co-cultured with unfractionated naïve CD4-expressing T cells at a ratio of 1:10. In agreement, the secretion of IL-17 was significantly enhanced when BIO5768-primed BMDCs were cocultured with CD4 T cells (Fig. 1D). To get further insights, cell surface markers of DC activation were analyzed by flow cytometry. Clearly, BIO5768 significantly increased cell surface presence of MHCII (p < 0.01), CD40 (p < 0.05) and CD86 (p < 0.01), demonstrating a strong effect of BIO5768 on the maturation of DC (Fig. 1E). Collectively, these experiments revealed that BIO5768 support the capacity of DCs to induce the production of IL-17 and IL-22 by CD4^+^ T cells and type 3 innate lymphoid cells, respectively.

### IL-17 signaling is required for induction of Angiogenin-4 in response to *L. acidophilus* BIO5768 independently of NOD2

**Figure 2:**
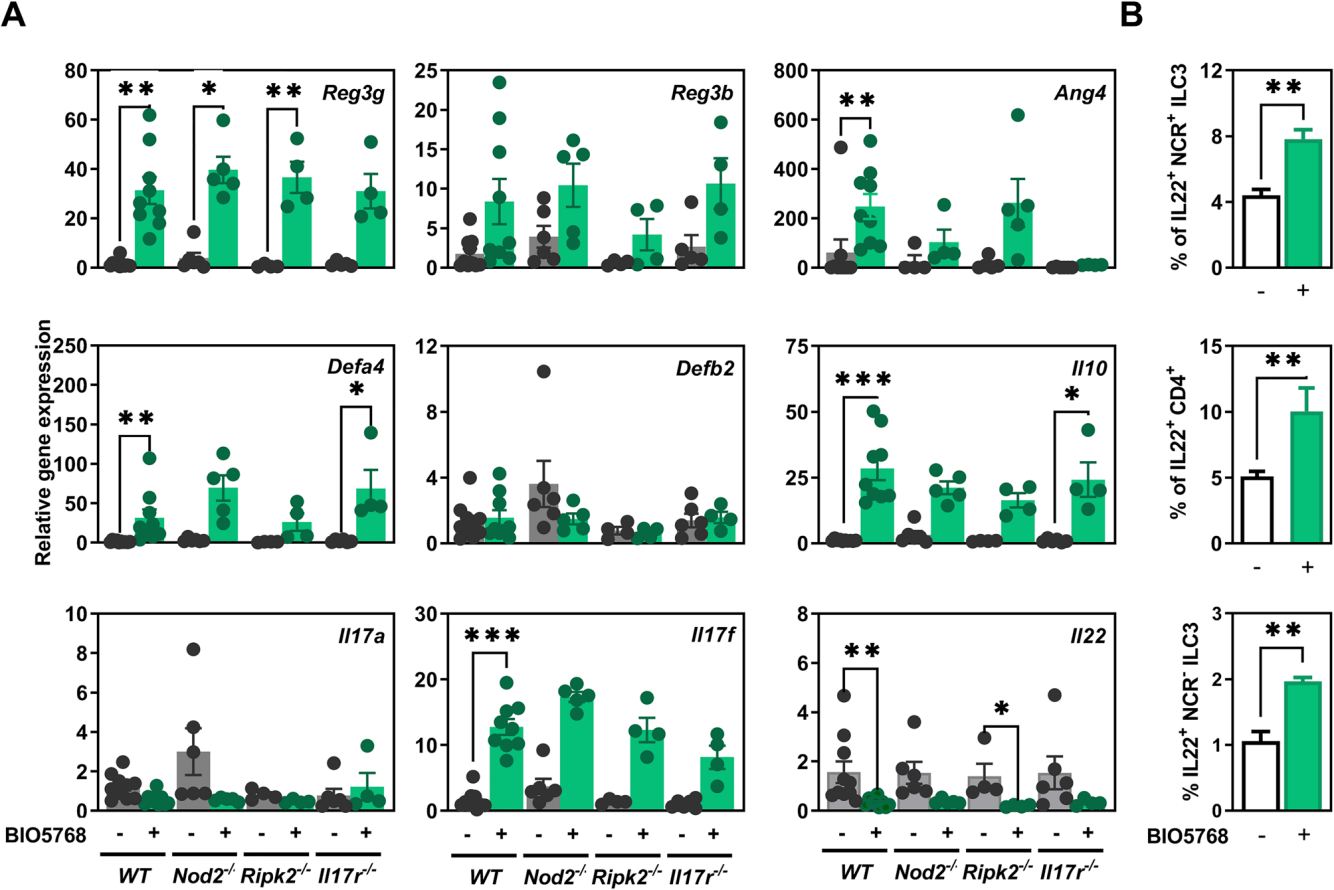
*The anti-microbial abilities of L. acidophilus* BIO5768 in vivo is independent of NOD2 and IL-17 signaling. GF female WT (n = 9) or deficient for NOD2 (*Nod2*^*−/−*^; n = 5), RIP2 (*Rip2*^*−/−*^; n = 4), and IL-17 Receptor A (*Il17Ra*^*−/*^; n = 4) were mono-colonized with strain BIO5768 by a single administration (5 × 10^8^ CFU/ mice) and compared to untreated GF WT mice (n = 9). (**A**) After 30 days mono-association, gene expression by qRT-PCR analysis of *Reg3g*, *Reg3b*, *Ang4*, *Defa4*, *Defb2*, *Il10*, *Il17a*, *Il17f* and *Il22* in the distal colon and caecum of monocolonized and GF mice. Results are expressed as means ± SEM by comparing the PCR cycle thresholds (Ct) for the gene of interest and for the house keeping gene Glyceraldehyde-3-Phosphate Dehydrogenase (*Gapdh)* of mono-colonized animals compared with GF WT mice. (**B**) Impact of BIO5768 monocolonization on the percentage of IL-22 producing NCR + and NCR− ILC3 and CD4^+^ T cells within colon and caecum, as determined by flow cytometry. Data represent means values of each group from one gnotobiotic experiment ± SEM. * p < 0.05; **p < 0.01; ***p < 0.001.

To test the possible dependence of NOD2 and IL-17 signaling on the induction IL-22-mediated immune response to BIO5768, germ-free (GF) mice deficient for Nod2 (*Nod2*^−/−^), for the receptor-Interacting Protein 2 (*Rip2*^−/−^) and for IL-17 Receptor A *(IL17ra*^−/−^) were mono-colonized or not by BIO5768 and compared to wild-type (WT) germ free (GF) animals. Thirty days after mono-colonization, BIO5768 significantly promoted gene expression of *Defa4*, *Reg3g* and *Ang4* in both caecum and colon of mono-colonized WT mice when compared to GF animals (Fig. 2A and Fig. S4). The increase of Reg-3γ was also confirmed at the protein level after immunohistochemistry staining of small intestinal section of Balb/c mice that were supplemented (or not) with BIO5768 (Fig. S4). Of interest, the induction of *Ang4* expression was blunted in the colon of mono-colonized *Il-17r*^*−/−*^ mice, while greater variability was noticed for either similarly treated *Nod2*- or *Rip2*-deficient mice (Fig. 2A). Even if we also observed some heterogeneity between *Nod2*-deficient mice, the supplementation with BIO5768 heightened the expression of the known Nod2-target gene *Defa4* independently of IL-17 signaling (Fig. 2A). In agreement with our gene expression analysis depicted in Fig. 1, BIO5768 administration failed to induce colonic expression of *Defb2*, *Il17a* and *Reg3b*. On the other hand, BIO5768 induced gene expression of key immunoregulatory cytokine *Il10* and *Il17f* in WT independently of NOD2 and IL-17 signaling (Fig. 2A). In line with this observation, Nod2 expression was dispensable for the ability of BIO5768 to promote the maturation of BMDCs (data not shown). Furthermore, administration of BIO5768 significantly lowered transcript level of *Il22* in WT (p < 0.01), *Nod2*^*−/−*^ (p = 0.057) and *Rip2*^*−/−*^ (p < 0.01) to a similar extent as what observed with commensal bacteria belonging to class *Clostridia* that modulate retinoic acid concentration in the gut^21^. By contrast to what observed at the transcriptional level, FACS analysis of the large intestine and the caecum from GF and monocolonized mice revealed an enhanced proportion of IL-22-producing ILC3 and CD4^+^ T cells despite similar mean fluorescence intensity of IL-22 staining and numbers of either ILC3 or CD4^+^ T cells (Fig. 2B). Collectively, these results indicate that sensing of BIO5768 indirectly modulates accumulation of IL-22-producing ILC3 in an IL-17 dependent manner.

### *L. acidophilus* BIO5768 downregulates inflammatory responses induced by *Citrobacter rodentium* infection

**Figure 3:**
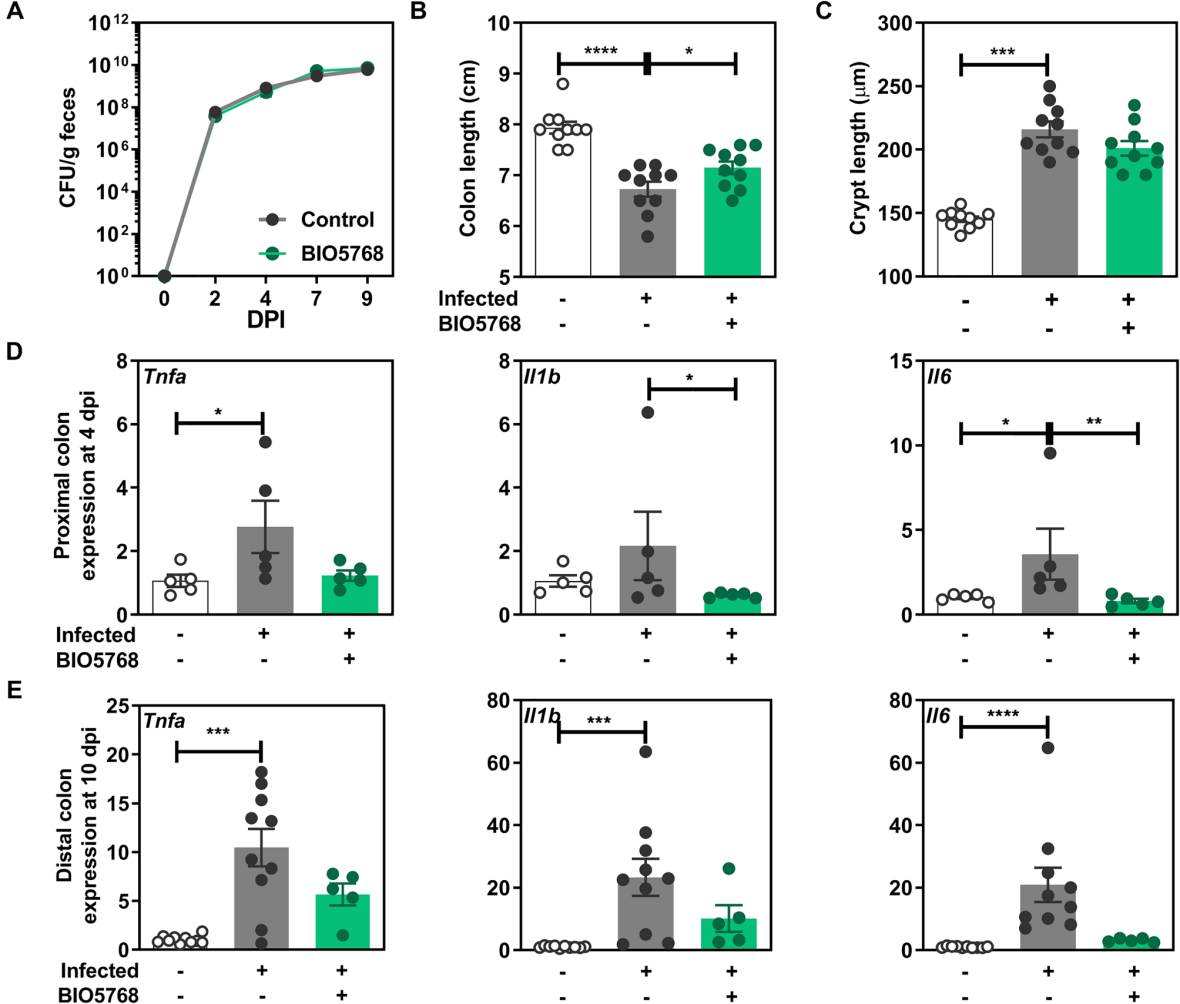
Impact of *L. acidophilus* BIO5768 supplementation in a mouse model of *C. rodentium* infection. BIO5768 (5 × 10^8^ CFU) was daily administered to C57BL/6J mice 5 days prior to infection and after the infection until the day of sacrifice. Mice were orally inoculated with 1 × 10^9^ CFU *C. rodentium* DBS 120 K strain and sacrificed at either 4 or 9 days after infection. (**A**) Effect of BIO5768 on bacterial load of *C. rodentium* determined by plating over the course of the experiment, fecal samples dilution on Luria Bertani medium containing 50 µg/ml kanamycin and compared with control infected mice. (**B**) Effect of BIO5768 supplementation on the colon length. (**C**) Effect of BIO5768 supplementation on the crypt length. (**D**) qRT-PCR analysis of the expression of pro-inflammatory genes in the proximal colon of mice after 4 days post infection. (**E**) qRT-PCR analysis of the expression of pro-inflammatory genes in the distal colon of mice after 10 days post infection. Results are expressed as relative gene expressions (2^−∆∆ct^) values, by comparing the PCR cycle thresholds (Ct) for the gene of interest and for the house keeping gene β-actin (*bact*), compared with control mice. Results of a representative experiment out of 2 are expressed as means ± SEM of uninfected control mice (n = 10) and infected mice that are supplemented (n = 5) or not (n = 10) with BI05768. * p < 0.05; ** p < 0.01; *** p < 0.001; **** p < 0.0001.

A *Citrobacter rodentium* infection model in mice was used since both IL-17 and IL-22 are involved in host defense against this pathogenic bacterium. *C. rodentium* mimics the human situation in which enteropathogenic and enterohemorrhagic strains of *Escherichia coli* contribute to the development of intestinal diarrhea. We therefore evaluated the impact of the oral administration of BIO5768 on the prevention and/or limitation of transient colitis caused by this pathogenic bacterium. Despite no effect of BIO5768 on bacterial burden (Fig. 3A), administration of BIO5768 improved the colon shortening that is caused by *C. rodentium* (Fig. 3B). By contrast, no effect of BIO5768 administration was observed on crypts hyperplasia measured at day 10 post-infection (Fig. 3C). *C. rodentium* infection significantly increased the colonic expression of genes encoding for TNF-α and IL-6 at day four post infection compared to non-infected mice (p < 0.05). Administration of BIO5768 significantly improved the transcript level of *Il6* at the proximal colon of mice (p < 0.05) compared to group infected by *C. rodentium* without probiotic bacteria supplementation (Fig. 3D). After 9 days post infection, the expression of *Tnfa* (p < 0.001), *Il6* (p < 0.0001), *Il1b* (p < 0.001) and *Il10* (p < 0.05) was significantly heightened in the distal colon of infected mice, despite no difference on Foxp3, when compared to non-infected animals (Fig. 3E and Fig. S4). By contrast, the expression of these aforementioned pro-inflammatory markers in the colon of infected mice supplemented with BIO5768 was similar to the one of non-infected animals (Fig. 3E).

### The mixture of BIO5768 with two other strains at least partially alleviated inflammation in the *C. rodentium* infection model and promotes accumulation of IL-22-producing type 3 innate lymphoid cells in a NOD2-dependent manner

**Figure 4:**
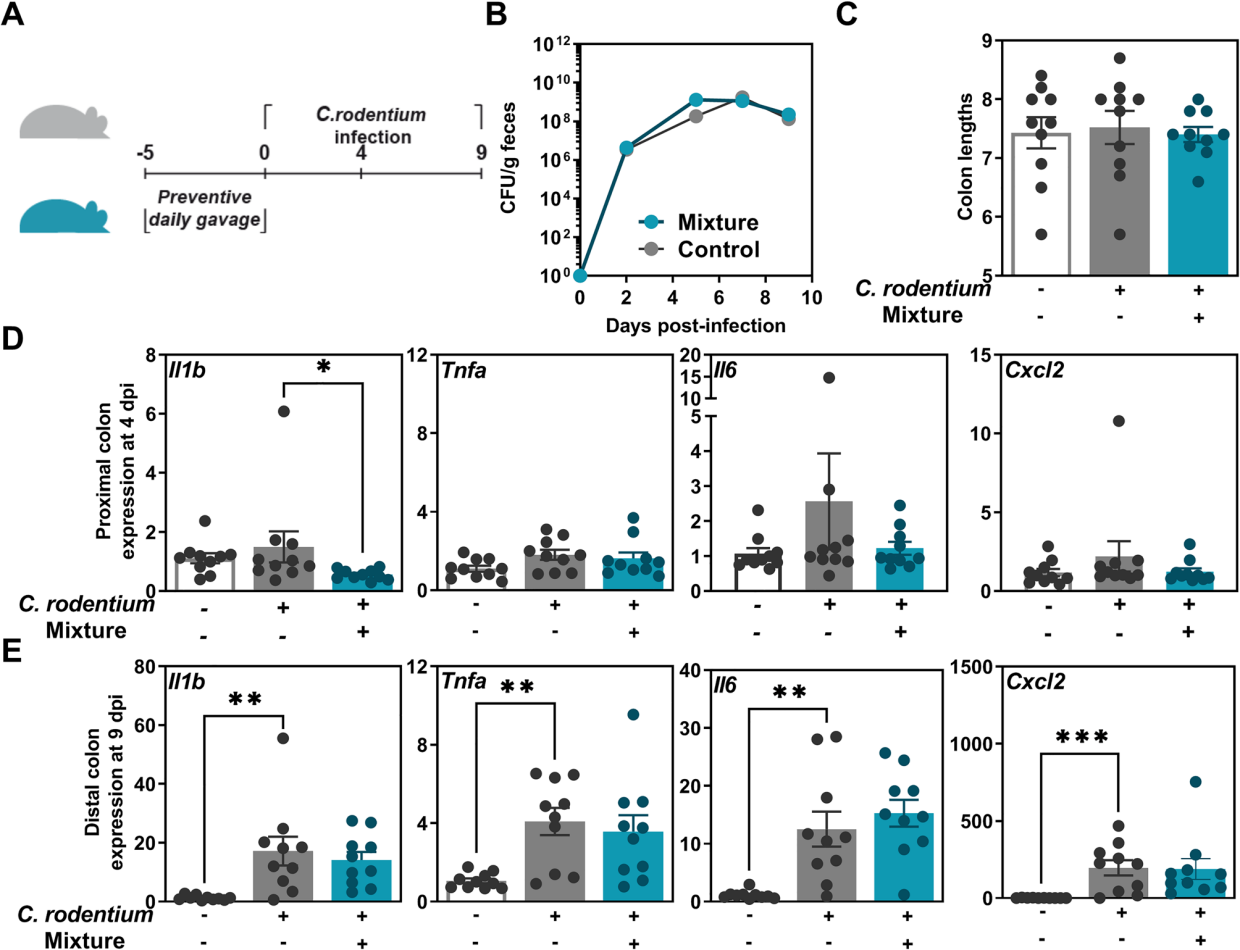
Capacity of the mixture to limit *Citrobacter rodentium*-induced colitis in C57BL/6J mice. (**A**) Experimental design. Mixture of three bacterial strains (5 × 10^8^ CFU/day/mice, all strains were present in equal amount in the mixture) was daily administered by intragastric preventive gavage for 5 days (n = 10 per group). (**B**) *C. rodentium* burden, (**C**) Colon lengths. (**D**) qRT-PCR analysis of the expression of pro-inflammatory genes in the proximal colon of mice after 4 days post infection when compared to non-infected animals. (**E**) qRT-PCR analysis of the expression of pro-inflammatory genes in the distal colon of mice after 10 days post infection when compared to non-infected animals. Results from one experiment are expressed as relative gene expressions (2^−∆∆ct^) by comparing the PCR cycle thresholds (Ct) for the gene of interest and for the house keeping gene β-actin (*bact*), compared with control mice. * p < 0.05; ** p < 0.01, *** p < 0.001.

We evaluated the capacity of *L. acidophilus* BIO5768 in combination (referred as mixture) with the two other strains *B. animalis* spp. *lactis* BIO5764 and *Li. reuteri* BIO5454 that were previously shown to limit the severity of colitis caused by *C. rodentium*^20^. Mice were treated orally for five consecutive days by the mixture prior to *C. rodentium* infection (Fig. 4A). Similarly to single strain application (for BIO5764 and BIO5454 see reference^20^ and for BIO5768 see Fig. 3A), the mixture had no effect on neither the burden of *C. rodentium* (Fig. 4B) nor on the colon length (Fig. 4C). However, the mixture supplementation was able to significantly downregulate the gene expression of *Il1b* (p < 0.01—Fig. 4D) as what observed with single strain administration (Fig. 3D). By contrast, only moderate effect of the infection was measured on the colonic expression of either *Cxcl2*, *Tnfa* or *Il6* at day 4 post-infection (Fig. 4D). After 9 days post-infection, the amplitude of the inflammatory response to the infection was significantly enhanced when compared to earlier time point (Fig. 4E). In addition, the induction of the aforementioned genes was not observed upon the supplementation of the mixture to the mice that were subsequently infected when compared to non-infected mice (Fig. 4E). As what observed with BIO5768 alone, the mixture was not able to increase significantly the abundance of CD4^+^ CD25^+^ FoxP3^+^ Tregs in MLN (Fig. S6), as previously observed with BIO5464^20^. Daily administration of the mixture to WT mice modestly induced the transcript level of *Il22* in a NOD2/RIP2-dependent manner, although there was no change in expression of *Defa4*, which is a NOD2-induced antimicrobial peptides (Fig. S6A,B). The capacity of the mixture to promote accumulation of IL-22-producing ILC3 isolated from MLN was then confirmed by multiparameter flow cytometry analysis. As what observed with BIO5768 alone (Fig. 1D), the mixture significantly elevated IL-22 production by NCR^−^ ILC3 in WT mice but not in either *Nod2*-or *Rip2*-deficient mice (Fig. S6C). In agreement with our in vitro data that are depicted in Fig. 1, we failed to detect any change in the proportion of IL-17-producing CD4 T cells at steady state (Fig. S6D). Given that BIO5768 has the capacity to promote the accumulation of IL-22-producing ILC3 independently of NOD2, the *Bifidobacterium* strain BIO5764 that belongs to the phylum of *Actinobacteria* is likely responsible for the NOD2-dependent IL-22 response to the mixture.

### *L. acidophilus* BIO5768 ameliorates the severity of TNBS-induced acute colitis in mice

**Figure 5:**
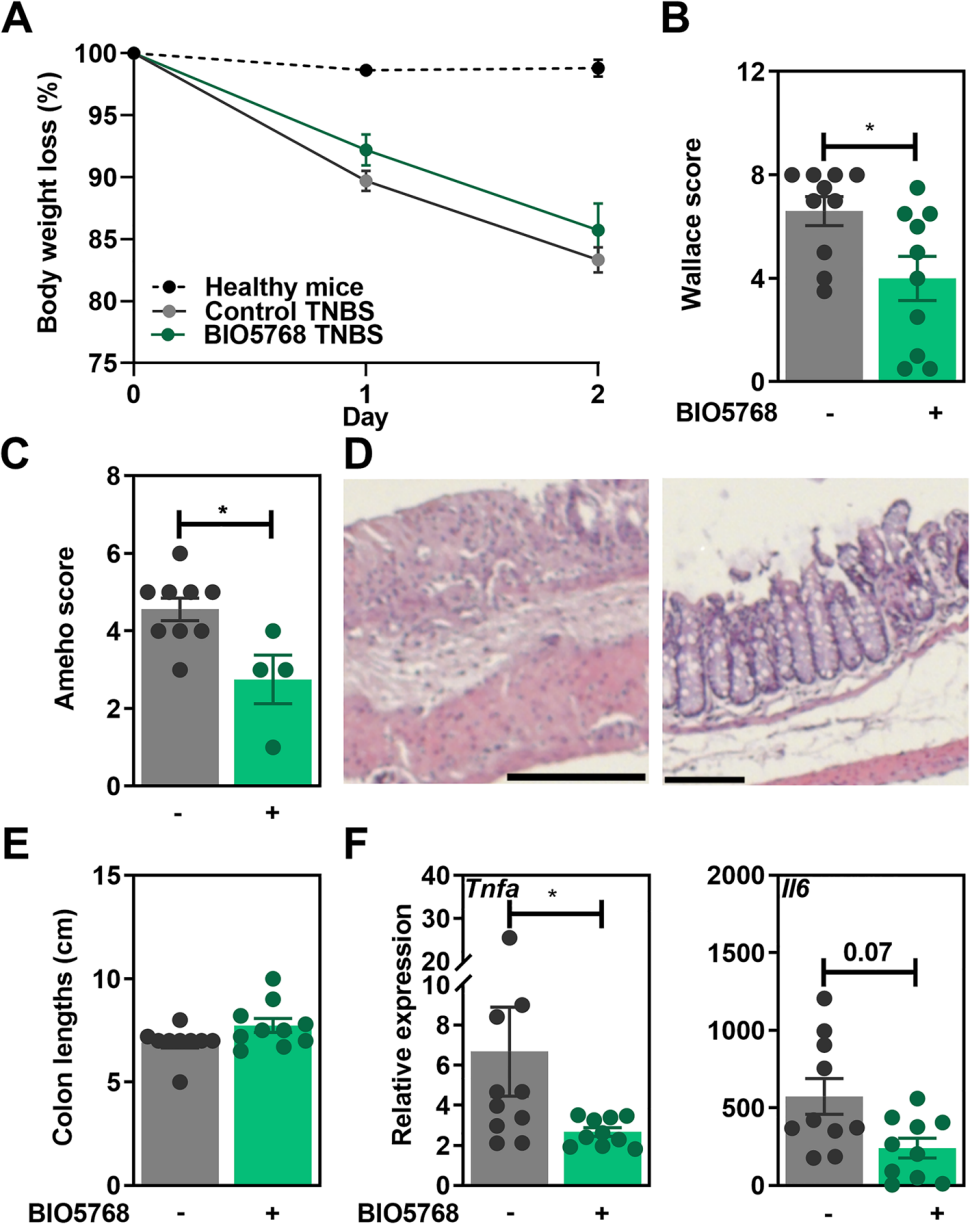
Protective effect of *L. acidophilus* BIO5768 supplementation in a mouse model of TNBS-induced colitis**.** BIO5768 (5 × 10^8^ CFU of each) was administered to BALB/c mice for 5 consecutive days before and 1 day after TNBS induction (n = 10 mice per group). After 48 h, mice were sacrificed and the impact of BIO5768 was analyzed on (**A**) Body weight loss, (**B**) Macroscopic grading of inflammation according to Wallace score, (**C**) Histological analyses of colon tissue according to Ameho score. (**D**) Representative histological sections (**E**) Colon lengths. (**F**) Gene expression of pro-inflammatory markers as determined by qRT-PCR analysis. Results are expressed as relative gene expressions (2^−∆∆ct^) values, by comparing the PCR cycle thresholds (Ct) for the gene of interest and for the house keeping gene β-actin (*bact*), compared with control mice. Results correspond to means ± SEM of 10 mice per group of a representative experiment from 2 experiments. *p < 0.05.

The potential ability of the strain BIO5768 to limit the severity of colitis was first evaluated in an experimental murine model of TNBS-induced acute colitis. While we observed only a moderate effect of the bacterial oral supplementation on weight loss (Fig. 5A), BIO5768 administration dampened the severity of colitis as indicated by a significant decrease of the macroscopic Wallace score (Fig. 5B; p < 0.05), confirmed by the histological analyses of colon tissue indicated by a decreased Ameho score, albeit at the limit of significance (Fig. 5C,D; p = 0.06). Despite no differences in the colon lengths (Fig. 5E), the expression of genes encoding the pro-inflammatory markers *Tnfa* and *Cxcl2* was also significantly (p < 0.05) downregulated in the BIO5738-treated group and to a lower extend also the gene encoding for *Il6* (Fig. 5F).

## Discussion

The anti-inflammatory potential of different *Lactobacilli* and *Bifidobacteria* strains in has been largely reported by use of either chemically -induced colitis or infectious colitis^22–26^. Given that we previously reported that the anti-inflammatory capacity of selected lactobacilli relied on an intact NOD2 signaling^18^, we postulated that the NOD2-dependent beneficial effect of probiotics could explain the failure of clinical trials using probiotics in CD. In agreement with our hypothesis, successful results were observed in ulcerative colitis, in which NOD2 plays a minor role if any^14,17^. Genome-wide association studies unveiled that *NOD2* is the most important genetic factor linked to abnormal dendritic cell function and AMP production in ileal CD, which are a largely reported feature of the disease. Therefore the selection of probiotic strains able to exhibit protective effects in a NOD2-independent manner is relevant to alleviate CD outcome in patients carrying *NOD2* mutations.

*Lactobacillus acidophilus* is one of the main commercial species of lactic acid bacteria available in different types of dairy products or dietary supplements with claimed probiotic effects^25^. Interestingly, *L. acidophilus* was reported to suppress the activation of the IL-23/Th17 axis associated with DSS-induced colitis^26^. RORγt is involved in Th17 cell development, which produces the key effector cytokine IL-17, playing a dual role in IBD. Th17 cells have been shown to be capable of regulatory functions and to be crucial in maintaining mucosal immunity against specific pathogens by promoting mucosal barrier repair. In this report, we demonstrated the capacity of the strain *L. acidophilus* BIO5768 to improve the severity of inflammation in two experimental models of colitis. The beneficial effect of this facultative anaerobic bacterium was partly mediated by its capacity to promote the functional activity of NCR- subset of ILC3 to secrete IL-22. Furthermore, we demonstrate that BIO5768 may promote the maturation of dendritic cells for supporting the production of IL-17 by CD4 T cells. Consequently, the epithelial expression of transcripts encoding for IL-22 and downstream target gene was blunted in *Il17ra*^−/−^ mice. This led us to provide evidence that the selected strain BIO5768 triggers IL-17-mediated AMP expression independently of NOD2 signaling. This potential probiotic strain could be of great interest for the treatment of CD patients with loss-of-function NOD2 polymorphisms. In agreement with our observation, neonatal and adulthood supplementation of mice by *L. acidophilus* also limited the severity of pathology induced by *C. rodentium* in mice^16,27^. Similarly, *L. acidophilus* NCFM was shown to be effective in inhibiting colitis induced by *C. rodentium*^28^ and other strains are also able to induce a sustained AMP production even if it remains to be determined whether it occurs independently of NOD2^29^. Further studies on the efficacy of this bacterial strain in preclinical models of chronic colitis is now awaited, especially in models exhibiting NOD2 deficiency. Indeed, the NOD2fs mutation results in a loss-of-function phenotype in human myeloid dendritic cells in CD^1^. To confirm the efficacy of the strain to treat patients with NOD2 mutations, fate mapping studies are also needed to assess probiotic-induced trained immunity including the migration capacity of DC in vivo.

A strain-dependent effect among probiotic species is well known, and differences among *L. acidophilus* strains in improving intestinal epithelial barrier function was reported recently^30^. Notably, the strain of *L. acidophilus* LA1 uniquely enhanced intestinal tight junction^31^ barrier function in a TLR2 dependent manner^30^. *L. acidophilus* was also reported to exhibit antioxidant and anti-inflammatory potential in an experimental model of arthritis^32^. The protective effect of *L. acidophilus* against experimental colitis was also shown to be dose-dependent, emphasizing the importance of selecting an optimal dosing regimen^33^. A recent report also indicated that the anti-inflammatory abilities of *L. acidophilus* LA-5 depend on the matrix in which the bacterium is delivered (capsules versus yogurt)^34^. Different *Lactobacillus* related taxa and especially *Limosilactobacillus reuteri* were also shown to activate IL-22 production by ILC3^35,36^. Recently, a protective effect of *Li. reuteri* D8 on tissue repair has been documented in vitro using co-cultured system with *lamina propria* lymphocytes (LPLs) in an organoid model^37^. The authors demonstrated that the indole-3-aldehyde produced by the strain stimulated LPLs to produce IL-22 through Aryl hydrocarbon Receptor and subsequent phosphorylation of signal transducer and activator of transcription 3 (STAT3)^37^. Other studies have reported that supplementation with three *Lactobacillus* strains with high tryptophan-metabolizing activities were able to restore intestinal IL-22 production^38,39^. Lactobacilli were also reported to maintain healthy gut mucosa by producing L-ornithine able to increase the level of l-kynurenine and subsequent expansion of RORγt^+^ IL-22^+^ ILC3 cells^40^. On the contrary, Kennedy et al.^41^ observed no effect of *L. plantarum* 299 on TNBS-induced colitis in rat experimental model. It suggests that probiotics may mediate their beneficial effect in a strain-dependent manner via distinct signaling pathways that overall remain poorly studied.

Many studies reported improved performance of probiotic mixtures, compared to individual strains. A mixture containing *L. acidophilus* was shown to alleviate DSS-induced colitis, notably by increasing the expressions of tight junctions and by upregulating the number of Tregs^42^. We then evaluated its probiotic properties in combination with the two other strains *B. animalis* spp. *lactis* BIO5764 and *Li. reuteri* BIO5454, previously reported to exhibit anti-inflammatory abilities in experimentally induced colitis, albeit with different modes of action^20^. Indeed, strain BIO5454 efficiently triggered IL-22 secretion by ILC3 and CD4^+^ T in vitro, and induced regulatory lymphocytes and NOD2-independent AMP expression. BIO5764 efficiently induced IL-17A and IL-22 in a NOD2-dependent manner, while having a minor impact on Tregs^20^. In the *C. rodentium* infectious colitis model, the mixture with the three strains was able to downregulate the expression of inflammatory genes, in a similar manner as the BIO5768 strain alone. Combining of this strain with two other dairy strains maintained not only the anti-inflammatory potential, but the mixture promoted the secretion of IL-22 by NCR-negative subset of ILC3 in a NOD2-dependent manner.

Our results suggest that the selected BIO5768 strain alone could provide an interesting complementary therapy in maintaining remission and improving the quality of life of patients. It remains necessary to investigate their clinical efficacy and the safety of the strains even in CD patients bearing NOD2 mutations.

## Materials and methods

### Bacterial strains

The three bacterial strains were selected from the Bioprox collection: *Limosilactobacillus reuteri* BIO5454 (BIO5454), *Lactobacillus acidophilus* BIO5768 (BIO5768), *Bifidobacterium animalis* spp. *lactis* BIO5764 (BIO5764). Lactobacilli were grown at 37 °C in MRS broth (Difco, Detroit, USA). Bifidobacteria were cultured in MRS media supplemented with cysteine (0.5 µg/ml) under anaerobic condition. Bacteria were grown overnight, harvested by centrifugation (10 min at 4000 × g), washed twice with PBS buffer (pH 7.2). For in vitro stimulation, bacteria were resuspended in PBS containing 20% (v/v) glycerol to a final concentration of 2 × 10^9^ CFU/ml and stored at − 80 °C until used. For in vivo administration, fresh cultured bacteria were resuspended in PBS at 2.5 × 10^9^ CFU/ml and were intragastrically administrated to mice (5 × 10^8^ CFU in 200 µl) as described previously^20^.

### Mice

C57BL/6 and BALB/c female mice were purchased from Charles River (L´Arbresle, France) and were housed in specific pathogen-free condition in the animal facilities of the Institut Pasteur de Lille (accredited No. C59-350009). 7–8 week old mice were maintained in a temperature-controlled (20 ± 2 °C) facility with a strict 12-h dark/light cycle. Animal experiments were performed in compliance with European guidelines of laboratory animal care (number 86/609/CEE) and with French legislation (Government Act 87–848). The study was carried out in compliance with the ARRIVE guideline and was approved by local Animal Ethics Committees (Nord-Pas-de-Calais CEEA N_75, Lille, France) and the *Ministère de l’Education Nationale, de l’Enseignement Supérieur et de la Recherche*, France (accredited No. 201608251651940). For *Citrobacter rodentium* infection, experiments were performed in the biosafety level 2 facility as described previously^20^. Before experimentation, animals were provided a one-week acclimation period and were given ad libitum access to regular mouse chow and drinking water. GF that are deficient or not for Nod2 (*Nod2*^−/−^), for the receptor Interacting Protein 2 (*Rip2*^−/−^), and for IL-17 Receptor A (*Il17ra*^−/−^) were generated on a C57BL/6 background at TAMM/CNRS Orleans (TAAM agreement number: D-45-234-6) and were bred in isolators under strict GF conditions. Monocolonisation experiments were approved by national and local Animal Ethics Committees authorization number 1038.

### Preparation of bone marrow derived dendritic cells (BMDC)

BMDC were generated from bone marrow progenitor cells as described previously^43^. Briefly, progenitor cells were obtained by flushing tibia and femur from BALB/c mice and cultured at 37 °C under 5% CO_2_ in Iscove’s modified Dulbecco’s media supplemented by FCS (10%), gentamycin (50 µg/ml), glutamine (2 mM), β-mercaptoethanol (50 µM) and 10% of supernatant from a granulocyte–macrophage colony-stimulating factor (GM-CSF)-expressing J588 myeloma cell line for 10 days. On day 10, cells were stimulated by bacteria (ratio bacteria/cell: 10:1). After 24 h stimulation, BMDC were harvested, washed and stained using mAbs anti-CD11c PE-Cy7, CD40 PE, CD80 FITC, CD86 PE-Cy5, MHCII APC (eBioscience, San Diego, CA, USA) and acquired on BD FACS Canto II (Becton Dickinson).

### Preclinical model of TNBS-induced colitis

TNBS-induced murine model of acute colitis was performed using BALB/c mice as described previously^20^. Briefly, anesthetized mice received an intra-rectal administration of TNBS (Sigma-Aldrich Chemical, France; 110 mg/kg) dissolved in 0.9% NaCl/ethanol (50/50 v/v). The protective effect of the probiotic strain was evaluated by oral preventive administration (intragastric feeding) of bacteria (5 × 10^8^ CFU/mice) starting 5 days before colitis induction. Forty eight hours after colitis induction, mice were sacrificed and the severity of colitis was graded according to the macroscopic inflammation based on Wallace scoring method^44^. Histological analysis was performed on May-Grünwald-Giemsa stained 5 μm tissue sections from colon samples and inflammation was graded according to Ameho score. Immediately after sacrifice, colonic samples were taken and stored in RNAlater storage solution (Ambion, Austin, TX, USA) at − 80 °C until further processed.

### Preclinical model of infectious colitis

*Citrobacter rodentium* infection was performed using the kanamycin resistant DBS 120 K strain as described previously^20^. Briefly, a single colony of *C. rodentium* was cultured overnight in Luria Bertani broth containing 50 µg/ml kanamycin, under agitation. Bacteria suspension was centrifuged, washed, resuspended in PBS and adjusted to 5 × 10^9^ CFU/ml. Mice were infected by oral administration of *C. rodentium* (10^9^ CFU per mice). The potential capacity of the selected probiotic strains to limit inflammation caused by *C. rodentium* was evaluated upon intragastric administration of the bacteria or the mixture (5 × 10^8^ CFU per mice, all strains were present in equal amount in the mixture) 5 days prior *C. rodentium* infection and daily following infection until termination of the experiment. Mice were sacrificed 9 days after infection. Level of infection was monitored as described previously^20^. Histological analyses were performed on May-Grünwald-Giemsa stained 5 μm tissue sections from colon samples fixed in 10% formalin and embedded in paraffin and crypt length was measured using ZEN (Zeiss, Oberkochen, Germany). Immediately after sacrifice, proximal and distal colon segments were put in RNAlater® (Ambion, Life Technologies, Foster City, CA, USA) and frozen at − 80 °C until RNA extraction and qRT-PCR analysis.

### RNA extraction and analysis of gene expression using quantitative real-time polymerase chain reaction (qRT-PCR)

Intestinal samples were removed at sacrifice and stored in RNAlater® storage solution (Ambion, Life Technologies, Foster City, CA, USA) at − 80 °C until qRT-PCR analysis. Tissue samples were homogenized using Lysing Matrix D (MPbio, Eschwege, Germany) and total RNA from samples was extracted as described previously^20^. Briefly, Macherey-Nagel NucleoSpin RNAII isolation kit (Düren, Germany) was used for RNA extraction according to the manufacturer’s recommendation. RNA quantity and quality were checked by Nanodrop (260/280 nm, 260/230 nm) and 1 µg RNA was reverse-transcribed using the High Capacity cDNA Reverse Transcription Kit (Applied Biosystems, Woolston Warrington, UK). Quantitative RT-PCR (qRT-PCR) was performed using the Power SYBR Green PCR Master Mix (Applied Biosystems) on ViiA 7 Real-Time PCR System (Applied Biosystems). Primers sequences can be available upon request. Results are expressed as relative gene expressions (2^−∆∆ct^) values, by comparing the PCR cycle thresholds (Ct) for the gene of interest and for the house keeping gene β-actin (*bact*) (or Glyceraldehyde-3-Phosphate Dehydrogenase, *Gapdh* for mono-associated mice*)* as described previously^45^.

### Supplementation of naive mice and characterization of IL-22 and IL-17-producing cells and regulatory T cells in mesenteric lymph nodes and intestinal resection specimens by flow cytometry

Strains (5 × 10^8^ CFU/day/mice) were administered by intragastric gavage to WT conventional BALB/c mice for 5 days. Colon samples were removed at sacrifice and stored in RNAlater® storage solution (Ambion, Life Technologies, Foster City, CA, USA) at − 80 °C until qRT-PCR analysis. MLN and intestine were harvested and immediately processed for flow cytometry. Cell suspensions of MLN and intestine (3–5 × 10^6^ cells, in RPMI1640 supplemented by 10% FCS, 2 mM l-glutamine, 2 mM HEPES, 40 mg/ml gentamycine) were stimulated using the Leukocyte Activation Cocktail containing BD GolgiPlug (BD Biosciences) (1 μl/ml of cell suspension) for 5 h. Cells were stained by mAbs anti-mouse CD11c eFluor450, CD11b eFluor 450, B220 eFluor 450, CD3 eFluor 450, CD117 Alexa Fluor 700, NK1.1 PerCP-Cy5.5, NKp46 FITC (provided by eBioscience), CD4 APC-H7, CD90.2 BV 500; CD45RB BV 605, MHCII BV 650 (provided by BD Bioscience, san Jose, CA, USA). Subsequently, cells were permeabilized and fixed using the Transcription Factor Buffer Set (BD Bioscience), and intracellular staining was performed using mAbs anti-IL-17A eFluor450, anti-FOXP3 PE-Cy7, anti-IL-22 PE and RORgt APC (eBioscience). Flow cytometry data were analyzed using software FlowJo. Gating strategy and representative dot plots for control and BIO5768 treated mice are presented in Figs. S1 and S2.

### Monocolonisation of axenic mice with the BIO5768 strain

GF WT and *Nod2*^−/−^, *Rip2*^−/−^, and *Il17ra*^−/−^ mice (9–13 weeks old, C57BL/6 background) were mono-associated with the BIO5768 strain by a single intragastric gavage (5 × 10^8^ CFU/mice). After 30 days of mono-association, colon samples were removed at sacrifice and stored in RNAlater® storage solution (Ambion, Life Technologies, Foster City, CA, USA) at − 80 °C until qRT-PCR analysis was performed. Cell suspensions of colon and caecum from mono-colonized mice were performed as described previously^46^. Cells (1 × 10^5^ cells) were stained by mAbs anti-mouse CD11c FITC (HL3 clone), CD11b FITC (M1/70 clone), B220 FITC (RA3-6B2 clone), CD3 FITC (145-2C11 clone), NK1.1 FITC (PK136 clone), CD4 V500/Amcyan (RM4-5 clone), CD117 APC-H7 (2B8 clone), NKp46 APC/eFluor 660 (29A1.4 clone), Il7ra V450/PB (SB/199 clone), CD90.2 PE-Cy7 (53-2.1 clone). Mouse IgG2a, k (G155-178 clone) and Goat IgG (Poly5164 clone) were used as isotype controls. Subsequently, cells were permeabilized and fixed using the Transcription Factor Buffer Set (BD Bioscience) and intracellular staining was performed using mAbs anti-IL-22 PE (1H8PWSR clone) and RORgt PerCP-Cy5.5 (Q31-378 clone).

### Statistics

GraphPad Prism was employed for graph preparation and statistical evaluation. Statistical significance was determined using either non-parametric Mann Whitney test, non-parametric one-way analysis of variance (ANOVA) followed by Dunn multiple comparison posthoc test and non-parametric two-way ANOVA with Bonferroni post-tests (GraphPad Prism software). Data with p values ≤ 0.05 were considered to be significant. The Spearman correlation coefficient was used to analyze correlation between *Il22* and *Defb2* expression.

## Supplementary Information

Supplementary Figures.

## Data Availability

Except when specified data not shown that are available from the corresponding author on reasonable request, all data generated or analysed during this study are included in this published article and its supplementary information files.
