# Supplementary material for: Oral supplementation with selected Lactobacillus acidophilus triggers IL-17-dependent innate defense response, activation of innate lymphoid cells type 3 and improves colitis
Source: Sci Rep. 2022 Oct 20;12:17591. doi: 10.1038/s41598-022-21643-0 (PMC9585059; doi:10.1038/s41598-022-21643-0)
Supplement: Supplementary file 1 — Supplementary Figures. [file 41598_2022_21643_MOESM1_ESM.pdf]

Oral supplementation with selected *Lactobacillus acidophilus* triggers IL-17-dependent innate defense response, activation of innate lymphoid cells type 3 and improves colitis.

Jiří Hrdý[1,6] Aurélie Couturier-Maillard[2], Denise Boutillier[1], Carmen Lapadatescu[3], Philippe Blanc[3], Jan Procházka[4], Bruno Pot[1,7], Bernhard Ryffel[2,5, *], Corinne Grangette[1,*] & Mathias Chamaillard[1,8,*]

[1]Univ. Lille, CNRS, Inserm, CHU Lille, Institut Pasteur de Lille, U1019-UMR 9017 - CIIL - Centre d'Infection et d'Immunité de Lille, Lille 59000, France;
[2]INEM - UMR7355, Molecular Immunology, University and CNRS, Orleans, France and Institute of Infectious Diseases and Molecular Medicine (IDM), and Infectious Diseases,
[3]Bioprox, 7 rue Aristide Briand, 92300 Levallois-Perret, France
[4]Czech Centre for Phenogenomics, Institute of Molecular Genetics, Czech Academy of Sciences, 252 50 Vestec, Czech Republic.
[5]Division of Immunology and South African Medical Research Council (SAMRC) Immunology, Faculty of Health Sciences, University of Cape Town, Anzio Road, Observatory 7925, Cape Town, RSA and Department of Clinical Immunology;.
[6]Present address: Institute of Immunology and Microbiology, First Faculty of Medicine, Charles University, Prague, Czech Republic.
[7]Present address: Research Group of Industrial Microbiology and Food Biotechnology, Faculty of Sciences and Bioengineering Sciences, Vrije Universiteit Brussel, Brussels, Belgium.
[8]Present address: Univ. Lille, Inserm, U1003, F-59000 Lille, France
* Shared senior authorship.

Running title: *Lactobacillus acidophilus* alleviates colitis and activates ILC3 independently of NOD2.

Key words: angiogenins; Crohn’s disease; IL-22; innate lymphoid cells; probiotics.

Correspondence and requests for materials should be addressed to Mathias Chamaillard (mathias.chamaillard@inserm.fr) or to Corinne Grangette (corinne.grangette@pasteur-lille.fr)

Supplementary Figures

Supplementary Figure S1. Panel I Gating strategy of innate lymphoid cells type 3 (ILC3) identification in control mice (left panel) and BIO5768 treated mice (right panel). Proportion of ILC3 NCR (natural cytotoxicity receptor)+ and NCR- subpopulations were identified by gating on the lineage negative cells A), followed by a gating on the $NKp46^{-}$ cells (ILC3 NCR^{-}) and $NKp46^{+}$ (ILC3 NCR+), B) and selection of the cells double positive for the transcription factor RORgt and the cytokine IL-22 C) (i.e. $IL\text{-}22^{+}NCR^{-}ILC3$) and D) (i.e. IL-22+NCR+ILC3). C) ILC3 NCR- were negative for NKp46 (NKp46-) and positive for characteristic transcription factor of ILC3 $RORgt^{+}$, NCR-ILC3 were further inspected for their capacity to produce IL-22 and D) ILC3 NCR+ were positive for NKp46, typical ILC3 transcription marker RORgt and further characterized according their capacity to release IL-22. **Panel II Gating strategy of IL-22 secreting $CD4^{+}$ T cells in control mice (left panel) and BIO5768-treated mice (right panel).** Th17 cells were identified by gating on the cells double positive for the lineage marker and CD4+ followed by a gating on the cells double positive for A) the transcription factor RORgt characterizing Th17 CD4+ T cell subset and B) the cytokine IL-22.

Supplementary Figure S2. Gating strategy for all three subsets of innate lymphoid cells type 3 (ILC3) identification. Forward and side scatter area was used to define the lymphoid population A). LTi cells (lymphoid tissue inducers) were identified by gating on the lineage negative cells (FITC negative) B) followed by gating on the population double positive for the transcription factor RORgt and the cytokine IL-22. C) The other subsets of ILC3, NCR^{+} and NCR^{-} subpopulations were identified from lineage negative cells based on their NKp46 cell surface expression D) NKp46- cells were further analyzed according to the presence of RORgt transcription marker characterizing ILC3 and their capacity to secrete IL-22 E). NKp46 positive cells were further screen for the presence of the transcription factor RORgt and F) cytokine IL-22.

Supplementary Figure S3. Percentage of IL-22 producing NCR+ ILC3 and of Tregs cells within mesenteric lymph nodes of mice that were supplemented with BIO5768 (n=7) when compared to control animals (n=6), as determined by flow cytometry.

Supplementary Figure S4. Representative immunohistochemistry staining of Reg3β in the small intestine of BALB/c mice that were supplemented (or not) with BIO5768 or the mixture.

Supplementary Figure S5. qRT-PCR analysis of the gene expression of *Il-10* and *Foxp3* in the distal colon of mice after 10 days post infection. Results are expressed as relative gene expressions ($2^{-\Delta\Delta ct}$), by comparing the PCR cycle thresholds (Ct) for the gene of interest and for the house keeping gene encoding for β-ACTIN (*bact*), compared with control mice. * $p < 0.05$.

Supplementary Figure S6. NOD2-dependent effect of the mixture on antimicrobial peptides gene expression, on IL-22 production by innate lymphoid cells type 3 (ILC3) and LTi cells and on expansion of CD4⁺ CD25⁺ FoxP3⁺ regulatory T cells. *L. acidophilus* BIO5768 or the mixture containing the three bacterial strains ($5x10^8$ CFU/ day/ mice, all strains were present in equal amounts in the mixture) were administered by intragastric gavage for 5 days to either naïve C57BL/6J WT mice (n=8), *Nod2*-deficient mice (n=5) or *Rip2*-deficient mice (n=8). A) Gene expression in the distal colon of *Defb2, Defb4*, *Reg3b* and *Il22* evaluated by qRT-PCR analysis at steady state. B) Gene expression in the proximal colon of *Defb2, Defb4*, *Reg3b* and *Il22* evaluated by qRT-PCR analysis at steady state. Results from one experiment are expressed as relative gene expressions ($2^{-\Delta\Delta ct}$) values, by comparing the PCR cycle thresholds (Ct) for the gene of interest and for the house keeping gene β-actin (*bact*), compared with control mice. C) Impact of the mixture supplementation on IL-17 production by $CD4^+$ T cells and IL-22 production by the different subsets of ILC3 isolated from MLN and on the number of $CD4^+$ $CD25^+$ $FoxP3^+$ regulatory T cells. Data represent means values of each group ± SEM. * $p < 0.05$.

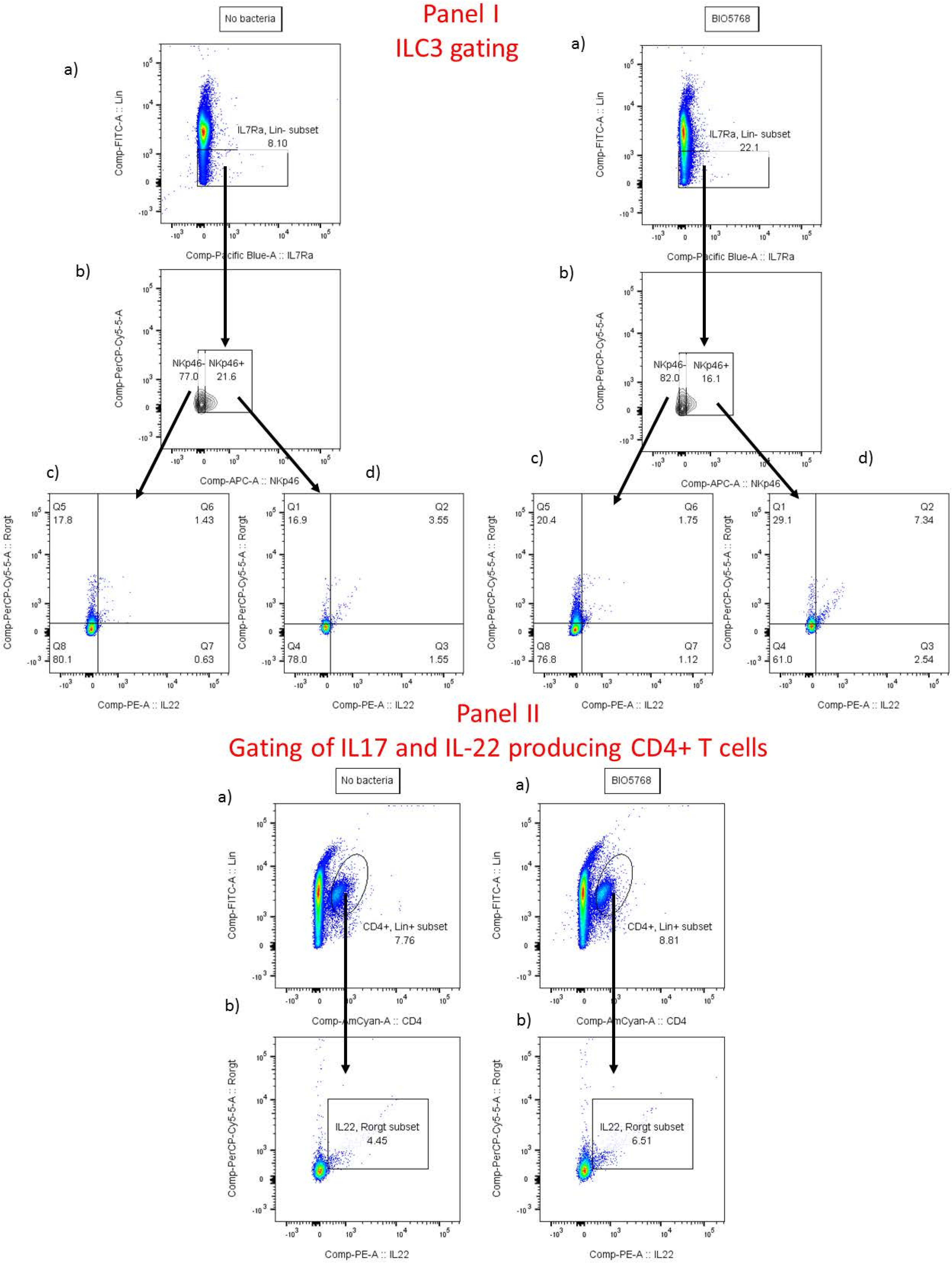

Panel I
ILC3 gating
No bacteria
BIO5768
a)
Comp-FITC-A :: Lin
Comp-Pacific Blue-A :: IL7Ra
IL7Ra, Lin- subset
8.10
22.1
b)
Comp-PerCP-Cy5-5-A
Comp-APC-A :: NKp46
NKp46-
77.0
NKp46+
21.6
NKp46-
82.0
NKp46+
16.1
c)
d)
Comp-PerCP-Cy5-5-A :: Rorgt
Comp-PE-A :: IL22
Q5
17.8
Q6
1.43
Q8
80.1
Q7
0.63
Q1
16.9
Q2
3.55
Q4
78.0
Q3
1.55
Q5
20.4
Q6
1.75
Q8
76.8
Q7
1.12
Q1
29.1
Q2
7.34
Q4
61.0
Q3
2.54
Panel II
Gating of IL17 and IL-22 producing CD4+ T cells
No bacteria
BIO5768
a)
Comp-FITC-A :: Lin
Comp-AmCyan-A :: CD4
CD4+, Lin+ subset
7.76
8.81
b)
Comp-PerCP-Cy5-5-A :: Rorgt
Comp-PE-A :: IL22
IL22, Rorgt subset
4.45
6.51

Gating strategy for all subsets of innate lymphoid cells identification

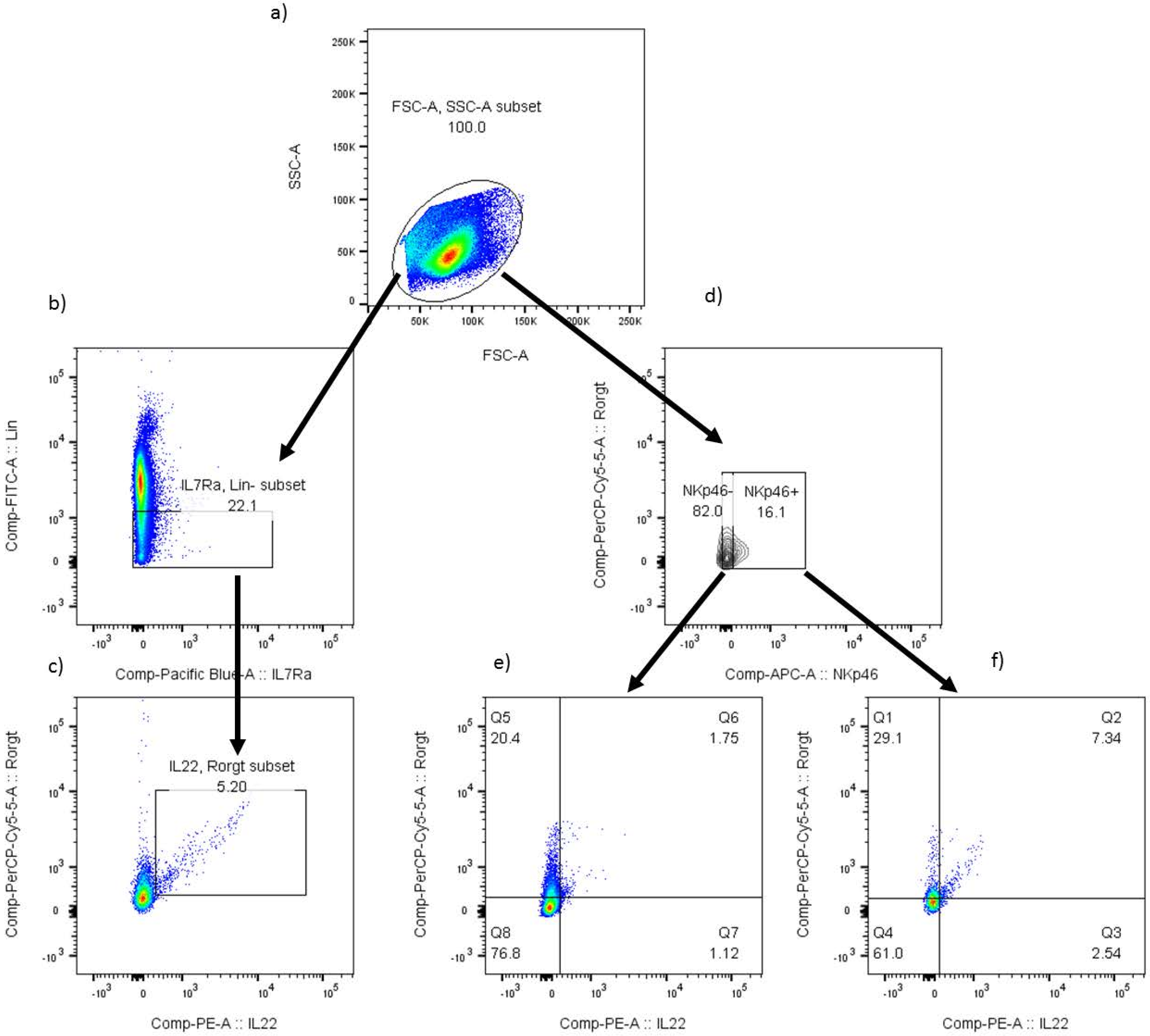

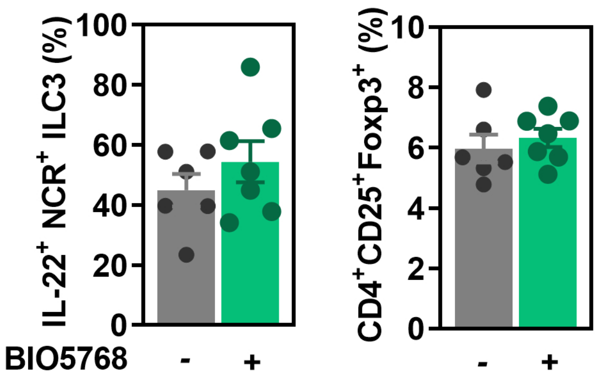

IL-22+ NCR+ ILC3 (%)
100
80
60
40
20
0
BIO5768
-
+
CD4+CD25+Foxp3+ (%)
10
8
6
4
2
0
-
+

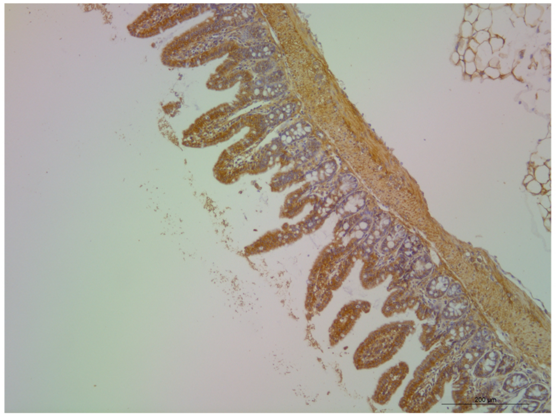

Control

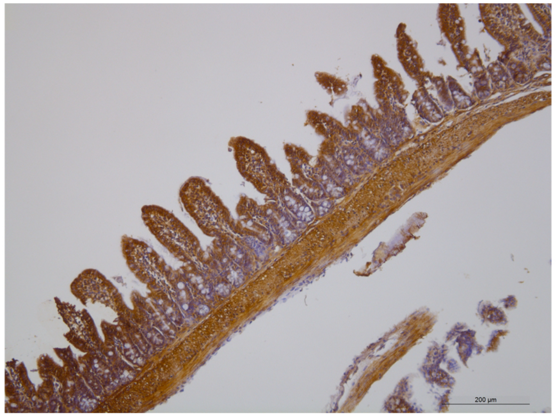

BIO5768

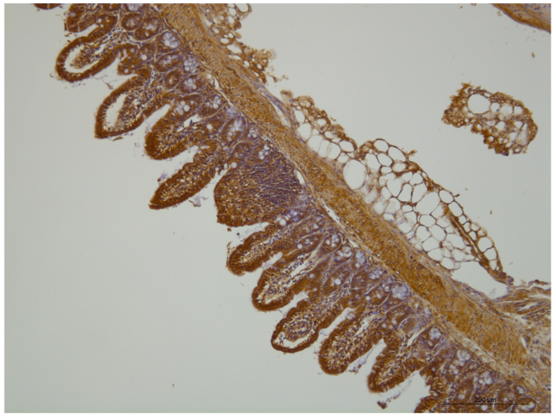

Mixture

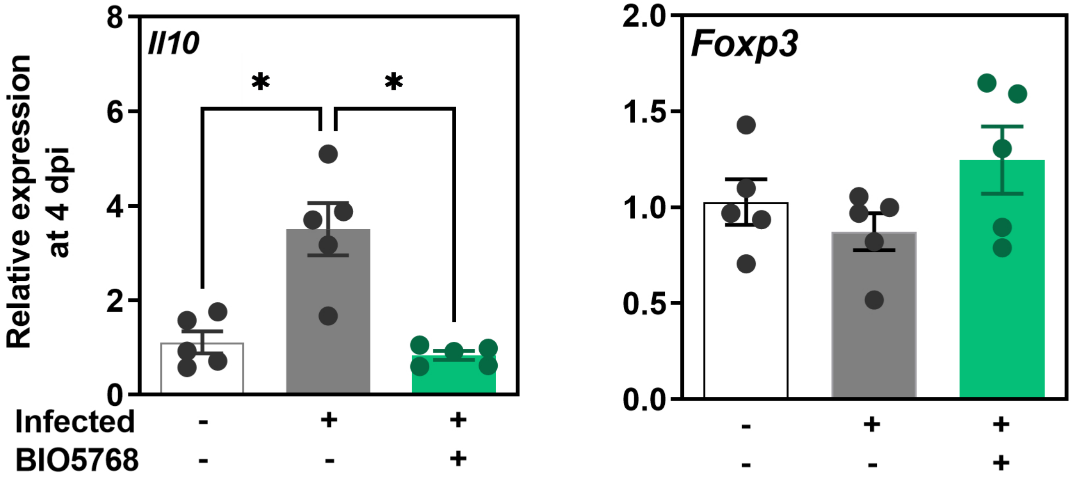

Relative expression at 4 dpi
Il10
Foxp3
8
6
4
2
0
2.0
1.5
1.0
0.5
0.0
*
*
Infected - + +
BIO5768 - - +

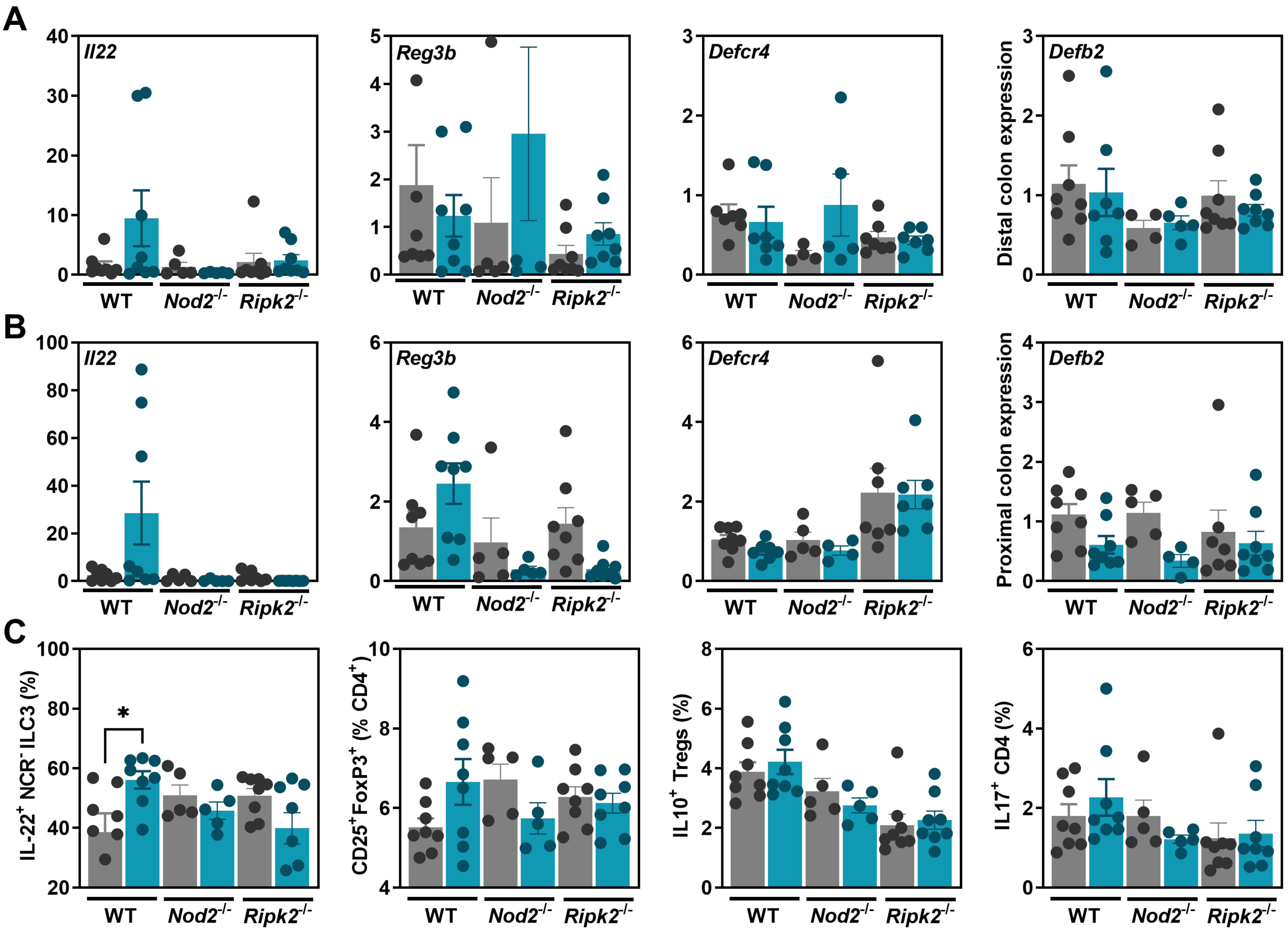

A
Il22
Reg3b
Defcr4
Defb2
Distal colon expression
WT
Nod2-/-
Ripk2-/-
B
Il22
Reg3b
Defcr4
Defb2
Proximal colon expression
WT
Nod2-/-
Ripk2-/-
C
IL-22+ NCR- ILC3 (%)
*
CD25+FoxP3+ (% CD4+)
IL10+ Tregs (%)
IL17+ CD4 (%)
WT
Nod2-/-
Ripk2-/-
